# 

**Published:** 1909-02-27

**Authors:** 


### THE GRADUATES' MEDICAL SCHOOLS.

DIARY FOR THE WEEK MARCH 1 TO MARCH 6.

**LONDON THROAT HOSPITAL, Great Portland Street, W.**

**At 5 p.m.**
**Mar. 3,** Dr. Potter, **Submucous Resection of the Nasal Septum.**

**THE POST-GRADUATE COLLEGE, West London Hospital, Hammersmith, W.**

**At 10 a.m.**
**Mar. 1 and 4,** Surgical Registrar, **Demonstration.**
**Mar. 5,** Medical Registrar, **Demonstration.**
**At 12 noon.**
**Mar. 1,** Dr. Low, **Pathological Demonstration.**
**At 12.15 p.m.**
**Mar. 3 and 5,** Dr. Pritchard, **Practical Medicine.**
**At 5 p.m.**
**Mar. 1,** Mr. Dunn, **Cases of Eye Disease.**
**Mar. 2,** Dr. Pritchard, **Clinical Pathology.**
**Mar. 3,** Dr. Beddard, **Medicine.**
**Mar. 4,** Mr. Edwards, **Clinical Lecture.**
**Mar. 5,** Mr. Etherington-Smith, **Clinical Lecture.**

**ST. JOHN'S HOSPITAL FOR DISEASES OF THE SKIN, Leicester Square, W.C.**

**Mar. 4,** Chesterfield Lectures, **Fungous Diseases of the Skin.**

**THE THROAT HOSPITAL, Golden Square, W.**

**At 5.30 p.m.**
**Mar. 1,** Mr. Parker, **Acute Inflammatory Affections of the Larynx.**
**Mar. 4,** Dr. Powell, **Chronic Inflammatory Affections of the Larynx.**

**NATIONAL HOSPITAL FOR THE PARALYSED AND EPILEPTIC, Queen Sq., Bloomsbury, W.C.**

**At 3.30 p.m.**
**Mar. 2,** Dr. Collier, **Disorders of the Nervous System in Renal Disease.**
**Mar. 5,** Dr. Ormerod, **Hysteria.**

**LONDON SCHOOL OF CLINICAL MEDICINE, Seamen's Hospital, Greenwich, S.E.**

**At 3.15 p.m.**
**Mar. 2,** Mr. Carless, **Fracture of the Patella.**
**At 2.15 p.m.**
**Mar. 5,** Dr. Rose Bradford.

**THE HOSPITAL FOR SICK CHILDREN, Great Ormond Street, W.C.**

**At 4 p.m.**
**Mar. 4,** Mr. Collier, **Demonstration of Surgical Cases**

**MEDICAL GRADUATES' COLLEGE AND POLYCLINIC, 22 Chenies Street, W.C.**

**At 5.15 p.m.**
**Mar 2.** Dr. Graham Little, **The Treatment of Blemishes of the Face.**
**Mar. 3,** Mr. R. H. J. Swan, **Renal Tuberculosis.**
**Mar. 4,** Mr. Jackson Clarke, **Metatarsalgia and Allied Conditions.**

**NORTH-EAST LONDON POST-GRADUATE COLLEGE, Prince of Wales's General Hospital, Tottenham, N.**

**At 4.30 p.m.**
**Mar. 5,** Mr. R. P. Brooks, **Types of Keratitis: their Diagnosis and Treatment.**

### MEDICAL GRADUATES' COLLEGE AND POLYCLINIC.

The Annual General Meeting will be held at 22 Chenies Street, London, W.C., on Monday, March 1, at 5 o'clock p.m. As matters of the greatest importance will have to be considered at this meeting, the Council particularly request the attendance of all members and subscribers.

The Problem of a Pure Milk Supply. Issued by Nestlé's Milk Co., St. George's House, Eastcheap, London, E.C.

The proprietors of Nestlé's Milk have lately issued a small book, which contains a series of fifty accredited instances in which children placed upon this food have thriven beyond expectation. The first pages deal with the composition and food value of Nestlé's Milk, and contain extracts from medical works on dietetics and the management of children; while towards the end are many useful recipes and directions for the preparation and administration of Nestlé's Milk in the infant's dietary. There are circumstances, especially in the poorer districts of cities, under which it is impossible to secure pure fresh milk for the children; and medical men will find it convenient to have particulars of the composition and methods of use of the brand of condensed milk which has long established itself as the most reliable in the market. A detailed bacteriological report from the Public Health Laboratories of University College London is appended and states that Néstle's Milk is "singularly free from micro-organisms of all kinds; those found (few in number compared with ordinary milk) being resistant . . . but harmless forms."

